# Oligo- and dsDNA-mediated genome editing using a *tetA* dual selection system in *Escherichia coli*

**DOI:** 10.1371/journal.pone.0181501

**Published:** 2017-07-18

**Authors:** Young Shin Ryu, Sathesh-Prabu Chandran, Kyungchul Kim, Sung Kuk Lee

**Affiliations:** 1 School of Energy and Chemical Engineering, Ulsan National Institute of Science and Technology (UNIST), Ulsan, Republic of Korea; 2 School of Life Sciences, Ulsan National Institute of Science and Technology (UNIST), Ulsan, Republic of Korea; Imperial College London, UNITED KINGDOM

## Abstract

The ability to precisely and seamlessly modify a target genome is needed for metabolic engineering and synthetic biology techniques aimed at creating potent biosystems. Herein, we report on a promising method in *Escherichia coli* that relies on the insertion of an optimized *tetA* dual selection cassette followed by replacement of the same cassette with short, single-stranded DNA (oligos) or long, double-stranded DNA and the isolation of recombinant strains by negative selection using NiCl_2_. This method could be rapidly and successfully used for genome engineering, including deletions, insertions, replacements, and point mutations, without inactivation of the methyl-directed mismatch repair (MMR) system and plasmid cloning. The method we describe here facilitates positive genome-edited recombinants with selection efficiencies ranging from 57 to 92%. Using our method, we increased lycopene production (3.4-fold) by replacing the ribosome binding site (RBS) of the rate-limiting gene (*dxs*) in the 1-deoxy-D-xylulose-5-phosphate (DXP) biosynthesis pathway with a strong RBS. Thus, this method could be used to achieve scarless, proficient, and targeted genome editing for engineering *E*. *coli* strains.

## Introduction

The bacterial genome has been previously manipulated via double-stranded (ds) DNA homologous recombination or short single-stranded (ss) DNA (oligo)-mediated genome engineering to modify bacterial strains. Several tools have been developed and used, such as multiplex automated genomic engineering (MAGE) and related approaches [1], zinc finger nucleases (ZFNs) [2], transcription activator-like effector nucleases (TALENs) [3], and clustered regularly interspaced short palindromic repeat (CRISPR)-associated Cas9 nucleases [4]. With recent advances in synthetic biology and metabolic engineering, genome modification has become important for the production of desired strains in basic and applied research [5–10]. However, each method has disadvantages compared with the others in terms of recombination and selection efficiency, insertion size, host genotype, and plasmid cloning. Therefore, there remains a demand for simple and efficient methods that can be easily used for multi-purpose genome engineering.

Conventional tools exploit separate positive- and negative-selection marker genes. Antibiotic-resistant marker genes such as *neo*, *bla*, *cat*, and *tetA* [11–15] are used to select the recombinants, whereas *SceI*, *sacB*, *rpsL*, *tolC*, *galK*, and *thyA* [16–18] have been used to select for positive-marker, gene-free clones; PCR-generated mutations in these negative-selection markers lead to the loss of their critical function, resulting in the selection of false-positive clones [16–19]. A dual selection system using a single gene for both positive and negative selection is considered as a more promising strategy in selecting desired recombinants because of its double functionality and elimination of inactive-marker genes caused by non-specific mutations [15,20]. TetA exports tetracycline from bacterial cells, conferring resistance to the antibiotic tetracycline [21–23], and pumps cadmium [24] and nickel [25] cations into the cell, resulting in cell death. However, it has been reported that *tetA* negative selection is much less effective in *Escherichia coli* than in other bacteria such as *Salmonella* [22].

Recently, a scarless genome modification method, the CRISPR-Cas9 system, has been developed using a double strand break for selection rather than antibiotic resistance and negative-selection markers. This system has limitations and complications such as high escape frequency, off-site effects, cellular toxicity of nucleases, plasmid cloning, and the requirement of a protospacer adjacent motif (PAM) sequence (5′-NGG-3ʹ) for specific targeting and expression of large Cas9 proteins [26–28]. Moreover, it has been reported that point mutations in *cas9* or elsewhere in the guide RNA plasmid result in cells that “escape” CRISPR-induced death [27]. Because of the high escape frequency, large DNA insertions are very difficult to obtain with the CRISPR-Cas9 system. More recently, Tas et al. [29] developed a genome modification method for *E*. *coli* using counter selection markers (either *tetA* or *galK*) in association with the λ-Red proteins SceI and RecA. In addition to high mutation efficiency, this method has disadvantages such as expression of multiple proteins and use of expensive chemicals and medium components to achieve maximum efficiency.

In this study, we developed a *tetA*-based dual selection system in *E*. *coli* for both short oligo- and long dsDNA-mediated genome editing. This method could be rapidly and successfully used for genome engineering without plasmid cloning. We demonstrated the efficacy of our system by optimizing metabolic flux through the 1-deoxy-D-xylulose-5-phosphate (DXP) biosynthesis pathway. We suggest that this system could achieve scarless, efficient targeted genome editing with a seamless screening process.

## Materials and methods

### Bacterial strains, plasmids, and oligomers

Wild-type *E*. *coli* MG1655 (MG) was used as the host strain for genome editing. The bacterial strains and plasmids as well as the oligomers used in this study are listed in Tables 1 and 2, respectively. Oligonucleotides were synthesized by Macrogen (Seoul, Korea).

**Table 1:** *Escherichia coli* strains and plasmids used in this study.

Strains/plasmids	Description/genotype	References/sources	
**Strains**			
MG	*E*. *coli* K-12 MG1655 (F^–^λ^–^*ilvG*^–^*rfb-50rph-1*)		[30]
MG-Red	MG with pSIM5		This study
MG-PT	MG Δ*lacZ*::P_*tetA*_-*tetA*^a^		This study
MG-PC	MG Δ*lacZ*::P_*cp25*_-*tetA*^a^		This study
MG-PP3	MG Δ*lacZ*::P_*P3BCD2*_-*tetA*^a^		This study
MG-LacZ0	MG *lacZ*_838-839_::P_*P3BCD2*_-*tetA*^b^		This study
MG-LacZ40	MG Δ*lacZ*_838-879_::P_*P3BCD2*_-*tetA*^b^		This study
MG-LacZ1500	MG Δ*lacZ*_838-2299_::P_*P3BCD2*_-*tetA*^b^		This study
MG-eDXS	MG *dxs* with a strong 5'-UTR^c^ (_*eng*_5'-*UTR*)		This study
MG-Lyco	MG with pINZ-1-LYC04		This study
MG-eDXS−Lyco	MG-eDXS with pINZ-1-LYC04		This study
MG-DXS-FLAG	MG *dxs-*FLAG^d^		This study
MG-eDXS-FLAG	MG _*eng*_5'-*UTR*^c^*-dxs-*FLAG^d^		This study
MG-DXS-GFP	MG *dxs-gfp*^e^		This study
MG-eDXS-GFP	MG _*eng*_5'-*UTR*^c^*-dxs-gfp*^e^		This study
**Plasmids**			
pSIM5	pSC101-ts *ori*, Cm^r^, expressing λ-Red *gam*, *exo*, and *bet*		[31]
pBBR1MCS3	rep *ori*, Tc^R^		[32]
pINZ-1-LYC04	pSC101-ts *ori*, Kan^r^, expressing *Erwinia herbicola crtEBI* and *Hematococcus pluvialis ipi*		[33]

^a^The *lacZ* gene was completely replaced with the *tetA* dual selection cassette.

^b^The numbers indicate the site of insertion of the *tetA* dual selection cassette.

^c^The original 5'-UTR sequence (AACAATAAGTATTAATAGGCCCCTG) of the *dxs* gene was replaced with a designed strong UTR (CTAGTTGGTAAGGAGTCTATAGTTG).

^d^The 1 × FLAG tag was inserted in-frame before the stop codon of Dxs.

^e^The C-terminal Dxs and the N-terminal GFP were linked by the flexible sequences (encoding 2 × GGGGS) [34].

**Table 2:** Primers and oligos used in this study.

Name	Sequence
P_tetA_-F	TATGTTGTGTGGAATTGTGAGCGGATAACAATTTCACACAGGAAACAGCTGTGTAGGCTGGAGCTGCTTCGCAATTCTCATGTTTGACAGC
tetA-R	ATGGATTTCCTTACGCGAAATACGGGCAGACATGGCCTGCCCGGTTATTAATTCCGGGGATCCGTCGACCTCAGGTCGAGGTGGCCCGGC
P_CP25_-F	GTGTAGGCTGGAGCTGCTTCGCTTTGGCAGTTTATTCTTGACATGTAGTGAGGGGGCTGGTATAATCACATAGTACTGTTCACACAGGAAACAGCTATGAAATCTAACAATGCGCTC
P_P3BCD2_-F1	GCTGTGTAGGCTGGAGCTGCTTCGAAAAAATTTATTTGCTTATTAATCATCCGGCTCGTATAATGTGTGGAGGGCCCAAGTTCACTTAAAAAGGAGATCAACAATGAAAGCAAT
P_P3BCD2_-F2	GCTGTGTAGGCTGGAGCTGCTTCGAAGGAGATCAACAATGAAAGCAATTTTCGTACTGAAACATCTTAATCATGCTAAGGAGGTTTTCTAATGAAATCTAACAATGCGCTCATC
lacZ-H1P1	TATGTTGTGTGGAATTGTGAGCGGATAACAATTTCACACAGGAAACAGCTGTGTAGGCTGGAGCTGCTTCG
lacZ-P1F	AGGTCGCCAGCGGCACCGCGCCTTTCGGCGGTGAAATTATGTGTAGGCTGGAGCTGCTTC
lacZ-P4R	ACGTAGTGTGACGCGATCGGCATAACCACCACGCTCATCGATTCCGGGGATCCGTCGACC
P4R	CTGTCAAACATGAGAATTAA
lacZ-rescue 1	CCGCGCCTTTCGGCGGTGAAATTATCGATGAGCGTGGTGGTTATGCCGATCGCGTCACACTACGTCTGAACGTCGAAAACCCGAAACTGT
lacZP4-R2	GGCGTCAGCAGTTGTTTTTTATCGCCAATCCACATCTGTGATTCCGGGGATCCGTCGACC
lacZ-resF	GCATTTTTACGCGCCGGAGAAAAC
lacZ-resR	CATCAACGGTAATCGCCATTTGAC
dxs-H1F	CTACATCATCCAGCGTAATAAATAAACAATAAGTATTAATGTGTAGGCTGGAGCTGCTTCG
dxs-H2R	GGGTCGGGTATTTGGCAATATCAAAACTCATCAGGGGCCTATTCCGGGGATCCGTCGACC
UTR-dxs	GCTAGCGGACTACATCATCCAGCGTAATAAATACTAGTTGGTAAGGAGTCTATAGTTGATGAGTTTTGATATTGCCAAATACCCGACCCT
dxs-seqF	ACCAGCAACTTGGTAAAAGTACC
dxs-seqR	CGATTTTGTCGCGGCG
dxs-H1F2	TGCCGCTGGTATGGAAGCCAAAATCAAGGCCTGGCTGGCAGTGTAGGCTGGAGCTGCTTCG
dxs-H2R2	GAATAATTTCTTAAGCATAGCAGGAGTGGAGTAGGGATTAATTCCGGGGATCCGTCGACC
dxs-FLAG	GGAAGCCAAAATCAAGGCCTGGCTGGCAGATTACAAGGATGACGATGACAAGTAATCCCTACTCCACTCCTGCTATG
dxs-seqF2	GGTACGCTGATGCCAGAAGCGGCG
dxs-seqR2	GGGACGGCTGCTTTCTTCCGGCAG
dxs-gfpF	CGGCCTCGATGCCGCTGGTATGGAAGCCAAAATCAAGGCCTGGCTGGCAGGTGGTTGGTTCTGGTGGTGGTTCTATGAGTAAAGGAGAAGAACTTTTC
dxs-gfpR	TAGAGTCTATGAATAATTTCTTAAGCATAGCAGGAGTGGAGTAGGGATTATTTGTAGAGCTCATCCATGCCATG

Sequences homologous to the target sites are underlined.

### Media and culture conditions

Bacterial strains were cultured in Luria-Bertani Miller broth (LB: 10 g of NaCl, 5 g of yeast extract, and 10 g of tryptone per liter) at 37°C, whereas cells containing temperature-sensitive plasmids were cultured at 30°C. For oligo transformation, *E*. *coli* cells were cultured in LB Lennox broth (LB-L: 5 g of NaCl, 5 g of yeast extract, and 10 g of tryptone per liter). The medium was supplemented with suitable antibiotics for plasmid maintenance and selection of positive-antibiotic markers at the following concentrations: chloramphenicol (Cm) at 30 μg/mL or tetracycline (Tet) at 10 μg/mL. For the nickel selection process, the cells were cultured in liquid minimal medium containing M9 salts (6.78 g of NaH_2_PO_4_, 3 g of KH_2_PO_4_, 0.5 g of NaCl, and 1 g of NH_4_Cl per liter), 0.2% glucose, and 50 μM NiCl_2_. The cells were incubated in a shaking incubator at 200 rpm at 37°C or 30°C.

### Chromosomal *tetA* expression under different promoters

The *tetA* gene from pBBR1MCS3 was expressed on the *E*. *coli* chromosome under three different promoters: the *tetA* gene native promoter (P_*tetA*_) [32], the synthetic constitutive promoter CP25 (P_*CP25*_) [35], or the P3 promoter including a bicistronic ribosome binding site (RBS) termed P3-BCD2 (P_*P3BCD2*_) [36]. Construction of the dual selection cassettes containing *tetA* and the three different promoters is illustrated in S1 Fig, and the sequences of all three promoters are provided in S2 Fig. Briefly, *tetA* and P_*tetA*_ were amplified using the primers P_tetA_-F and tetA–R; P_*CP25*_ was linked to *tetA* using the primers P_CP25_-F and tetA–R; and P_*P3BCD2*_ was linked to *tetA* using three primers (i.e., P_P3BCD2_-F1, P_P3BCD2_-F2, and *tetA*–R) by overlap extension PCR. The same cassettes were PCR-amplified with primers lacZ-H1P1 and tetA–R and then integrated into the *lacZ* region of the MG-Red strain expressing the λ-Red recombinase genes (i.e., *gam*, *exo*, and *bet*) on pSIM5 [31] to construct the dual selection cassette variant strains MG-PT, MG-PC, and MG-PP3. The pSIM5 plasmid contains the temperature-sensitive replication protein RepA101, the λ-Red recombinase genes under the P_*L*_ promoter controlled by the cI857 temperature-sensitive repressor, and a chloramphenicol resistance marker gene. This plasmid can be cured simply by growing the cells at temperatures above 37°C and the λ-Red system can be easily switched on at 42°C and off at 32°C.

To identify the optimal NiCl_2_ and tetracycline concentrations for the dual selection process, the cell growth of the *tetA*-integrated strains (i.e., MG-PT, MG-PC, and MG-PP3) was monitored separately at different concentrations of NiCl_2_ (10−100 μM) and tetracycline (10−100 μg/mL). Cell growth was recorded by measuring absorption at 600 nm using a microplate reader (Infinite F200 PRO, Tecan) in a Corning 96-well, clear bottom plate with shaking (200 rpm, 37°C).

### Genome modification

For oligo-mediated genome editing, the dual selection cassette P_*P3BCD2*_-*tetA* was integrated either between 838 and 839 bp without deletion or between 838 and 879 bp with a 40-bp deletion of *lacZ* to generate two nonfunctional-*lacZ* strains, MG-LacZ0 and MG-LacZ40, respectively. A 90-nt oligo (i.e., lacZ rescue 1) flanked with sequences homologous to the target regions was designed to rescue the disrupted *lacZ* of the strains MG-LacZ0 and MG-LacZ40. For dsDNA-mediated genome editing, a 1.5-kb region in *lacZ* was deleted by the insertion of the P_*P3BCD2*_-*tetA*, resulting in the nonfunctional-*lacZ* strain MG-LacZ1500. The deleted region with 300 bp of homologous sequences on either side was PCR-amplified from intact *lacZ* using the primers lacZ–resF and lacZ–resR. To rescue the disrupted *lacZ* in the strains MG-LacZ0, MG-LacZ40, and MG-LacZ1500, the strains were transformed with the respective oligo or 2.1-kb PCR products. Briefly, the strain MG carrying the plasmid pSIM5 and P_*P3BCD2*_-*tetA* on the chromosome was grown in 5 mL of fresh LB-L containing Cm and Tet, at 30°C. The λ-Red system was induced at an optical density (OD_600_) of approximately 0.5 by heating at 42°C for 15−20 min in a water bath as previously described [31]. Cells were immediately chilled on ice for at least 20 min and then rendered electrocompetent. Oligonucleotides were added to 50 μL of cells at a final concentration of 5 μM and electroporation was performed using a MicroPulser electroporator (Bio-Rad). Following electro-transformation, the cells were recovered by 3-h outgrowth at 30°C in 3 mL of LB. The cells were then diluted 1:100 and sub-cultured in liquid M9 medium containing 0.2% glucose and 50 μM of NiCl_2_ at 37°C for approximately 16 h. To measure rescue/selection efficiency, the sub-cultured cells were diluted to 10^−6^ prior to being spread onto LB plates containing X-gal/IPTG, and the efficiency was calculated by counting the blue colonies from the total number of colonies. If required, a second round of recombination was performed: following the first round of electro-transformation, the 3 mL of cells recovered by 3-h outgrowth at 30°C were induced at 42°C for 15−20 min and subsequently transformed with the oligos or dsDNA. Selection in liquid M9 medium with NiCl_2_ was then performed as described above.

### Application to metabolic engineering

The P_*P3BCD2*_-*tetA* cassette was integrated into the 5′ untranslated region (5′-UTR) of *dxs*, which encodes one of the lycopene synthesis rate-limiting enzymes, in an *E*. *coli* strain expressing the λ-Red system on the plasmid pSIM5. A strong 5′-UTR (CTAGTTGGTAAGGAGTCTATAGTTG) was predicted using the UTR designer program [37]. The same cassette was then replaced with an oligo (i.e., UTR-dxs) containing a strong 5′-UTR flanked by the respective homologous sequences of *dxs*. Short ssDNA (oligos) transformation was performed twice and, after 3-h outgrowth, nickel selection was performed to obtain an engineered *dxs* strain, named MG-eDXS. The plasmid pINZ-1-LYC04, which contains heterologous genes required for the final steps of lycopene production [33,38], was transformed into MG and MG-eDXS resulting in the strains MG-Lyco and MG-eDXS-Lyco, respectively. For lycopene production, cells (i.e., MG-Lyco and MG-eDXS-Lyco) were cultured separately in 250-mL flasks containing 50 mL of LB broth at 30°C for 48 h. Lycopene was extracted with acetone and quantified by measuring absorbance (OD_470_). Lycopene content was calculated according to a standard curve as previously described [39]. To analyze DXS expression levels in MG-eDXS by Western blotting, a FLAG-tag sequence was inserted immediately after *dxs* in the strains MG and MG-eDXS resulting in the strains MG-DXS-FLAG and MG-eDXS-FLAG, respectively. Western blotting band intensity was measured using the ImageJ program (National Institutes of Health, USA). In addition, the FLAG-tag sequence was replaced in MG-DXS-FLAG and MG-eDXS-FLAG with *gfp*, which encodes green fluorescent protein (GFP), using our dsDNA homologous recombination genome editing method. This allowed determining *dxs* expression by measuring GFP intensity in the resultant variant strains MG-DXS-GFP and MG-eDXS-GFP, respectively. The genetic variants were confirmed by PCR and DNA sequencing.

## Results

### A new strategy for genome editing

**Fig 1:**
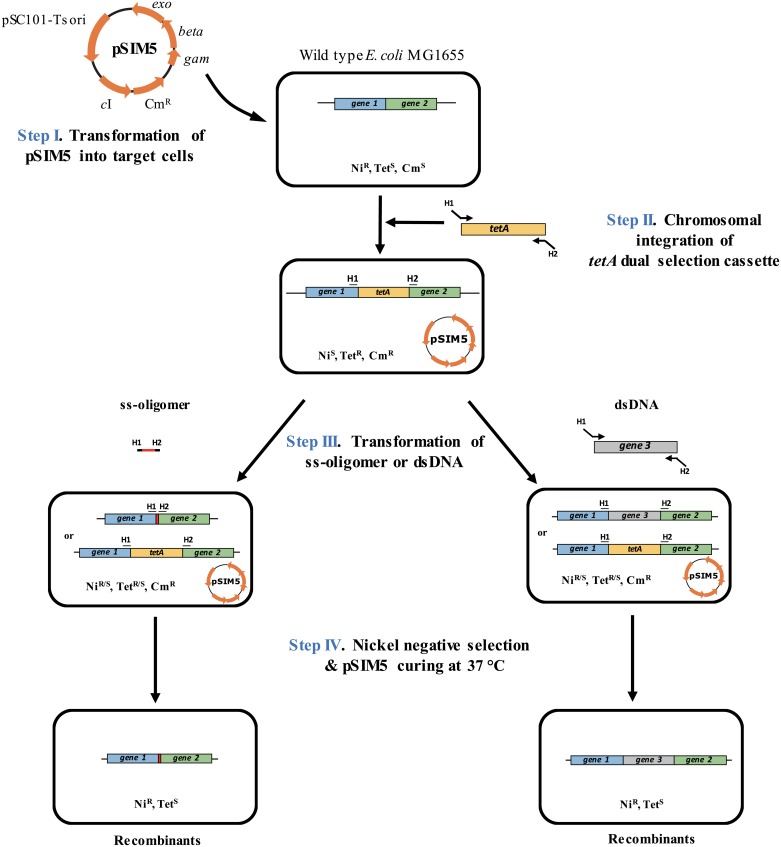
Schematic diagram of oligo- and dsDNA-mediated genome editing using a *tetA* dual selection system in *Escherichia coli*. Step I: Introduction of the λ-Red system into wild-type *E*. *coli*. Step II: Integration of the *tetA* dual selection cassette into the chromosomal target site. Step III: Genome editing by introduction of an ss-oligomer or dsDNA. Step IV: Selection of the genome-modified cells on NiCl_2_ under optimum selection conditions. A detailed description of this method is provided in the text.

The scheme of the proposed dual selection genome editing strategy is illustrated in Fig 1. Step I: the plasmid pSIM5 carrying the λ-Red system was transformed into the strain MG and the transformants were selected on an LB plate containing Cm (30 μg/mL) at 30°C. Step II: the dual selection cassette P_*P3BCD2*_-*tetA* flanked by 50-nt homologous sequences of the target region was PCR-amplified and transformed into an *E*. *coli* strain expressing the λ-Red system. Recombinant cells possessing the *tetA* dual selection cassette on the chromosome were selected on an LB plate containing Tet (10 μg/mL) at 30°C. Red recombineering functions were induced in cells carrying the plasmid pSIM5 by heating at 42°C for 15 min and thus made electrocompetent. Step III: ss-oligos or dsDNAs with homologous sequences were transformed into cells containing P_*P3BCD2*_-*tetA* on the target region of the chromosome and expressing the λ-Red system. The dual selection cassette was replaced by either oligo-mediated recombination or dsDNA homologous recombination. Step IV: the genome-modified recombinants were enriched by culturing the cells in M9 liquid medium containing NiCl_2_ (50 μM) at 37°C. The plasmid pSIM5 was simultaneously cured during this step. The cultured cells were streaked onto LB plates and the desired recombinants were confirmed by colony PCR and DNA sequencing. To obtain improved selection efficiency, additional selection can be performed to select non-edited cells containing *tet*A using replica plating of colonies from LB to both LB and LB-Tet plates. Colonies that grow on LB but fail to grow on LB-Tet are the desired mutants.

### Effective expression of chromosomal *tetA* for negative selection

**Fig 2:**
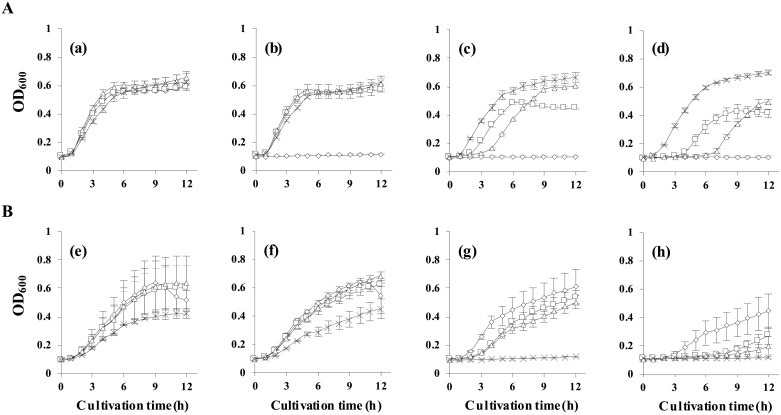
Promoter selection and optimization of NiCl_2_ and tetracycline concentrations for dual selection. *tetA* was integrated into the strain MG (◇) with three different promoters (i.e., P_*tetA*_, P_*CP25*_, and P_*P3BCD2*_) and the resulting variant strains MG-PT (□), MG-PC (Δ), and MG-PP3 (×) were grown in M9 medium containing different concentrations of tetracycline (A) [0 (a), 10 (b), 50 (c), and 100 (d) μg/mL] and NiCl_2_ (B) [0 (e), 10 (f), 50 (g), and 100 (h) μM] to identify the strongest promoter and the optimal concentrations of Tet and NiCl_2_ for the dual selection process. P_*P3BCD2*_ was strong enough to express the TetA protein and confer strong nickel sensitivity at 50 μM NiCl_2_ and tetracycline resistance at the highest concentration (100 μg/mL), thereby allowing successful selection of the target-edited mutants and elimination of target-unedited cells. Data represent the mean value of three independent experiments and error bars represent standard deviation.

Although the *tetA* negative-selection system is successful in *Salmonella*, it is much less effective in *E*. *coli* strains [22]. We hypothesized that TetA expression level is not sufficient for negative selection in *E*. *coli*. Thus, the relationship between the expression level of chromosomally encoded TetA and negative selection was tested by analyzing the growth of cells expressing *tetA* under different promoters (i.e., P_*tetA*_, P_*CP25*_, or P_*P3BCD2*_) at various concentrations of NiCl_2_ (up to 100 μM; Fig 2). The expression level of TetA under the three promoters was analyzed by measuring cell growth in the presence of high tetracycline concentration. Maximum growth was exhibited by P_*P3BCD2*_-*tetA* even at the highest concentration of tetracycline (100 μg/mL), indicating that *tetA* expression was sufficiently high to confer tetracycline resistance. All variants and wild-type strains were non-sensitive at 10 μM [Fig 2B(f)] and sensitive at 100 μM [Fig 2B(h)] NiCl_2_. Consistent with the tetracycline resistance results, when *tetA* was expressed under a known strong promoter, P_*P3BCD2*_, cell growth was significantly inhibited in the presence of NiCl_2_. At 50 μM NiCl_2_, wild-type cells could grow but cells expressing *tetA* under P_*P3BCD2*_ could not, indicating that negative selection is relatively effective at this concentration. The results indicate that both promoters (i.e., P_*tetA*_ and P_*CP25*_) were not strong enough to express *tetA* and to confer strong nickel sensitivity (Fig 2). In contrast, the P_*P3BCD2*_-*tetA* system could be used for *tetA* dual selection in *E*. *coli*.

### Selection efficiency of the *tetA*-based genome editing method

**Fig 3:**
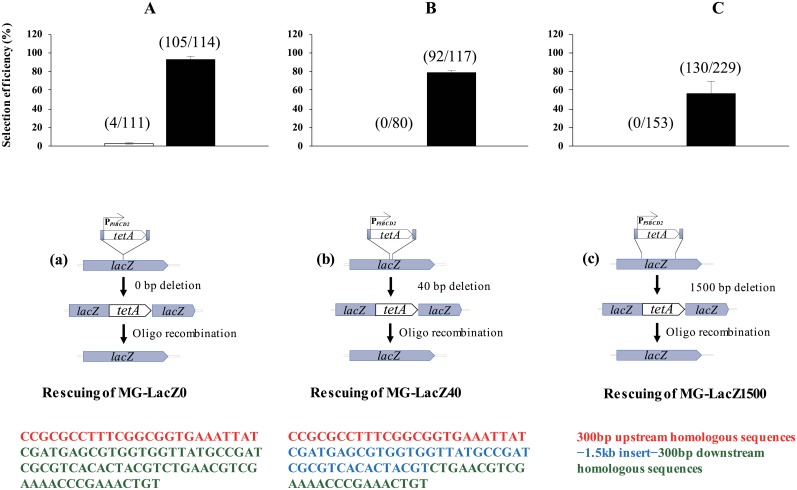
Genome editing using ssDNA oligos and linear dsDNA. The *lacZ* segment was targeted in our genome editing method. The *lacZ* region was disrupted by the integration of the dual selection cassette P_*P3BCD2*_-*tetA*, which was rescued with either an oligo, [MG-LacZ0 (A) or MG-LacZ40 (B)] or linear dsDNA [(MG-LacZ1500 (C)]. Filled and unfilled bars represent the selection efficiency obtained with and without nickel selection, respectively. Numbers above the bar indicate the number of blue colonies (numerator) and total number of colonies (denominator) obtained by spreading the transformants on an LB plate containing X-gal/IPTG. (a), (b), and (c) refer to the editing steps followed to achieve target genome editing. Red and green in the oligos sequences represent the regions homologous to *lacZ*. Blue sequences represent the inserted bases. Data represent the mean value of three independent experiments and error bars represent standard deviation.

The selection efficiency of the proposed genome editing method was determined using the *lacZ* region to perform common genetic modifications. The nonfunctional-*lacZ* strains (i.e., MG-LacZ0 and MG-LacZ40) were rescued by deleting the integrated dual selection cassette with oligo lacZ rescue 1. In the case of MG-LacZ0 rescue, selection efficiency for the functional *lacZ* following two recombination events was as high as 92% with nickel selection (Fig 3A), whereas, approximately 4% of the cells were identified as recombinants without nickel selection. This result indicated that, following oligo transformation, subculturing the transformants in liquid M9 salt medium containing NiCl_2_ increased the selection efficiency. The same trend was also found for MG-LacZ40 rescue. Moreover, no colonies with the desired insertions were obtained by selection in liquid M9 salt medium without NiCl_2_. The selection efficiency for functional *lacZ* rescue in the strain MG-LacZ40 following two recombination events approached 79% (Fig 3B). Approximately, 30- and 80-fold increase in selection efficiency was achieved in MG-LacZ0 or MG-LacZ40, respectively, by subculturing the cells in liquid M9 salt medium containing NiCl_2_, compared to subculturing in M9 salt medium alone. MG-LacZ0 showed 13% higher efficiency than MG-LacZ40 did because it contains a homologous region for recombination longer than that found in MG-LacZ40. Additionally, there was no significant difference in selection efficiency when two or more recombination events were carried out (data not shown). Subsequent rounds of genome modification are not obligatory and, moreover, considerable efficiency was obtained following only one recombination event.

The efficiency of our system for dsDNA-mediated recombination was also evaluated by rescuing the nonfunctional-*lacZ* strain, MG-LacZ1500, with a PCR-amplified dsDNA fragment (2.1 kb). The efficiency was approximately 57%, similar to that of the oligo-mediated method (Fig 3C). Furthermore, no colonies with the desired insertions were obtained by selection in liquid M9 salt medium without NiCl_2_, indicating that *tetA* negative selection is very effective in *E*. *coli*. The results suggest that our system is also suitable for long, linear dsDNA-mediated genome editing even in cases of very low integration efficiency. The widely known constraints of oligo-mediated insertions, including size (≤ 120 nt) [1], can be circumvented by our dsDNA-mediated recombination method. We successfully achieved linear dsDNA-mediated genome insertion of a *lacZ* fragment at least 1.5 kb in size with flanking homology regions of at least 300 bp.

### Application to metabolic engineering

**Fig 4:**
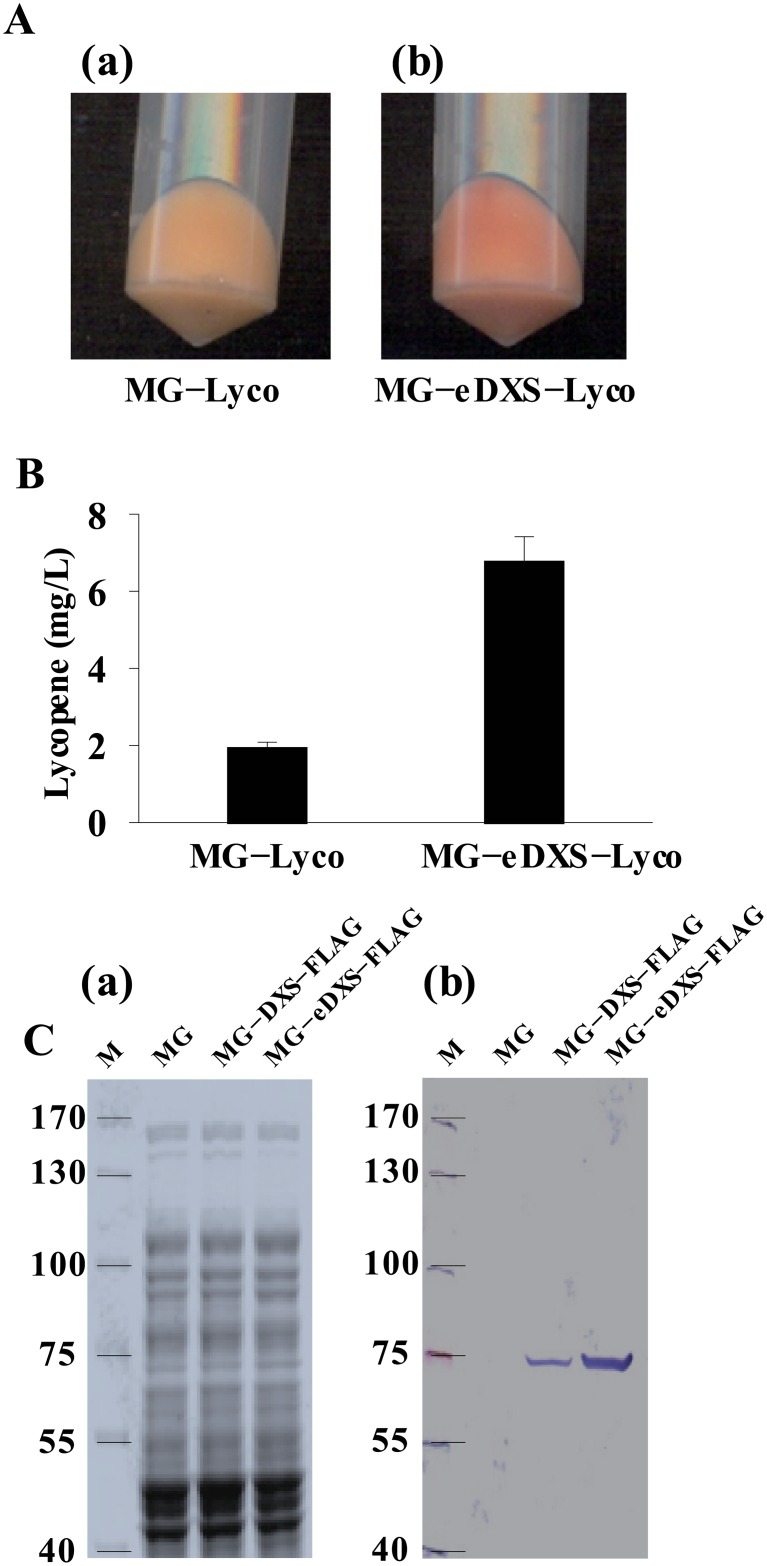
Application in metabolic engineering. Lycopene production was enhanced by increasing the translation efficiency of *dxs* in the lycopene synthesis pathway. The MG-eDXS-Lyco cell pellet [A(b)] showed intense red pigmentation compared to MG-Lyco [A(a)]. MG-eDXS-Lyco produced 3.4-fold lycopene amount than MG-Lyco did (B). *dxs* expression in MG-DXS-FLAG and MG-eDXS-FLAG was analyzed by SDS-PAGE [C(a)] and Western blotting [C(b)].

To demonstrate the applicability of our system, we optimized the metabolic flux through the DXP biosynthesis pathway to overproduce lycopene in *E*. *coli* MG1655 (MG). The 5′-UTR of *dxs*, the gene encoding the rate-limiting enzyme in lycopene biosynthesis, was replaced with a strong 5′-UTR to enhance the translation efficiency. The engineered strain MG-eDXS-Lyco produced intense red pigmentation caused by increased lycopene production [Fig 4A(b)] compared to MG-Lyco [Fig 4A(b)]. Lycopene production was 3.4-fold in MG-eDXS-Lyco compared to that in MG-Lyco (Fig 4B). To confirm the increase in *dxs* expression, a FLAG-tag sequence was inserted immediately after *dxs*, and the expression of Dxs-FLAG was analyzed by SDS-PAGE [Fig 4C(a)] and Western blotting [Fig 4C(b)]. Analysis of the relative Western blotting band intensity of MG-eDXS-FLAG and MG-DXS-FLAG showed that *dxs* expression level was highly increased (approximately 4-fold) in MG-eDXS-FLAG compared to MG-DXS-FLAG. Gene expression level can be measured by detecting the fluorescence intensity of *gfp* fused to the target gene. The *gfp* gene was integrated downstream of *dxs* using our method, resulting in the strains MG-DXS-GFP and MG-eDXS-GFP, which was confirmed by PCR and DNA sequencing (S3 Fig). However, GFP expression could not be detected, possibly due to low expression levels on the chromosome.

## Discussion

Successful selection of genome-edited recombinants requires high editing efficiency or high-throughput screening. One approach for increasing recombineering efficiency is λ-Red-assisted oligonucleotide-mediated recombination [40]. However, replacement with oligos containing more than five mismatched bases and the deletion of entire genes result in relatively low frequency of genomic modifications, hindering the selection of particular genetic variants from a large number of unedited colonies [41]. In addition, this method cannot be used for large dsDNA recombination with low efficiency because it does not include selection strategies. These limitations were overcome in our system by combining a *tetA*-based dual selection system with dsDNA homologous recombination or oligo-mediated recombination. Our system exhibits high selection efficiency, i.e., 57% and 92% for dsDNA and oligo-mediated recombineering, respectively.

Additionally, our method overcomes the problems of the popular *tetA*-*sacB* genome editing method, including the size of the *tetA*-*sacB* cassette, inefficiency of the counter-selection marker due to mutation in *sacB*, and the need for complex and expensive growth medium for counter-selection [15]. Mizoguchi et al. [42] reported that approximately 76% of the transformants presented false-positive phenotype when using the negative-selection marker *sacB* and the positive-selection marker *cat* due to mutations in the negative-selection marker gene *sacB*. The use of independent selection markers for positive and negative selections significantly increases the chances of the emergence of false positives due to the mutations in the negative selection marker. This problem has been addressed by the use of a single selection marker such as *tetA* as a dual selection marker because any mutation that affects the function of the dual selection marker protein will likely affect the both positive and negative selections [43,44], thus, significantly reducing the complications of false positives. In the *tetA* dual selection system, non-functional *tetA* mutants could be selected out during the first positive selection because the mutants cannot survive on an LB-Tet plate. The recently developed TetA/galK-SceI method takes 1−2 weeks from start to verified end-product [29]. The use of *galK* has disadvantages as the recipient host needs to be *galK*^−^, and counter-selection is performed using a medium with either an expensive galactose analogue, 2-deoxy-galactose, or low-cost NiCl_2_ [29]. Unlike other methods [1,15,27,29], our method can be performed rapidly at low cost in liquid minimal salt medium containing NiCl_2_. Our method accomplishes successful recombination in a short time frame (7−8 days) compared with other similar scarless genome editing methods such as CRISPR and SecI (S1 Table). Comparisons of our method with other popular scarless genome editing methods are detailed in Table 3.

**Table 3:** Comparison among popular genome editing methods.

Particulars	MAGE [1]	CRISPR [27]	*tetA*-*sacB* dual selection [15]	*tetA*-*SceI* dual selection [29]	*tetA* dual selection
dsDNA recombination	−	+	+	+	**+**
Oligo recombination	+	+	+	+	**+**
Recombinant selection	−	+	+^a^	+^a^	**+**^b^
MMR active	−	+	+	+	+
Plasmid cloning^c^	−	+	−	−	−
Mutation in selection system	NA	+^d^	+^e^	+^f^	− ^g^

^a^The counter-selection medium is complex and expensive.

^b^The counter-selection medium is simple and inexpensive.

^c^Plasmid cloning will take an additional week.

Possible mutation in *cas9*^d^, *sacB*^e^, and *SceI*^f^

^g^Non-functional *tetA* mutants could be selected out in the first positive selection because the mutants cannot survive on an LB-Tet plate.

NA—Not Applicable

The expression level of *tetA*, which confers metal sensitivity, is a key factor for employing the *tetA* dual selection system in genome editing applications [25,29,45]. Consistent with a previous study [22], nickel sensitivity for negative selection was not observed in cells harboring *tetA* on the chromosome under the control of either the native promoter P_*tetA*_ or the synthetic constitutive promoter P_*CP25*_, which is known to be suitable for ensuring strong expression in *E*. *coli* [35]. However, when *tetA* was expressed under P_*P3BCD2*_, a known strong promoter with a double RBS [36], nickel had toxic effects on cell growth in liquid M9 salt medium supplemented with 50 μM NiCl_2_. These findings indicate that *tetA* expression under P_*P3BCD2*_ was higher than under the other two promoters tested, and eventually resulted in nickel sensitivity, which might be due to sufficient expression of TetA for negative selection against nickel. We believe that the method proposed in this study could solve the problems linked to the *tetA* system and that it can be used effectively in *E*. *coli* for scarless and seamless genome editing.

Since dsDNA homologous recombination-mediated genome editing is much less efficient than the oligo-mediated method [46], target-edited cells were not obtained without the nickel selection step when a large dsDNA (2.1 kb) was inserted by homologous recombination. Following nickel negative selection, approximately 57% of the transformants were target-edited mutants, indicating that, although recombination efficiency was low, the genetic recombinants were enriched and the unedited cells were eliminated by nickel negative selection. In this study, we obtained high selection efficiency for both oligo-mediated and dsDNA homologous recombination under the optimized selection conditions. We suggest that this feature could be effectively employed for various genome-editing projects when selection or screening processes are unavailable.

Oligo-mediated genome engineering has high editing efficiency (maximum theoretical efficiency per recombination cycle is 25%) [1,41], producing many genome variants. Unlike dsDNA homologous recombination, this method does not include a selection process, resulting in difficulties in identifying specific recombinants, namely gene deletion and replacement of oligos presenting long mismatching sequences with low editing efficiency (< 0.1%). Low efficiency makes it difficult to select target recombinants from unedited cells. This problem can be solved by increasing the selection efficiency using the *tetA* dual selection system employed in our method.

## Conclusions

Here, we developed a reliable and versatile genome editing method by combining the novel application of oligo- or dsDNA-mediated genome editing and a *tetA* dual selection system that offers scarless genome modifications in *E*. *coli*. This method has additional advantages over other scarless genome editing tools. First, there is no need for plasmid cloning, thereby making it a simple, time-saving, and less labor-intensive method. Second, screening of recombinants by *tetA* dual selection renders the method very efficient as it reduces the number of false-positive mutants. Altogether, the method described herein is more robust than others and enables highly efficient manipulation of the bacterial genome, including the rewiring of metabolic pathways as well as the construction of new microbial cell factories for applied research and industrial applications.

## Supporting information

S1 Fig Construction of *tetA* dual selection cassettes with three different promoters. *tetA* amplified from pBBR1MCS3 was linked to three different promoters: the *tetA* gene native promoter (P_*tetA*_) [32], synthetic constitutive promoter CP25 (P_*CP25*_) [35], or the P3 promoter including a bicistronic ribosome binding site termed P3-BCD2 (P_*P3BCD2*_) [36], to construct the three different dual selection cassettes P_*tetA*_-TetA, P_*CP25*_-TetA, and P_*P3BCD2*_-TetA, respectively. These cassettes were individually transformed into MG-Red to construct dual selection cassette-variant strains, MG-PT, MG-PC, and MG-PP3. Detailed information is provided in the text.
(PDF)

S2 Fig Nucleotide sequences of the promoters used for chromosomal *tetA* expression in the *lacZ* region. (A) P_*tetA*_: native promoter of *tetA* [32]; (B) P_*CP25*_: synthetic constitutive promoter CP25 [35]; (C) P_*P3BCD2*_: synthetic constitutive promoter P3 including bicistronic ribosome binding site [36]; P1 and P4: priming sequences [47]; H1 and H2: upstream and downstream homologous sequences; −35 and −10: promoter regions; +1: transcription start site.
(PDF)

S3 Fig Gene insertion using dsDNA-homologous recombination. (A) Agarose gel electrophoresis of the PCR products following the insertion of *gfp* downstream of *dxs* (B) Steps and primers (dxs-seqF2 and dxs-seqR2) used to confirm recombination. The size of specific PCR products is given in parentheses.
(PDF)

S1 Table Timeline of the workflow for the *tetA* dual selection. (PDF)
