# Supplementary material for: Oligo- and dsDNA-mediated genome editing using a tetA dual selection system in Escherichia coli
Source: PLoS One. 2017 Jul 18;12(7):e0181501. doi: 10.1371/journal.pone.0181501 (PMC5515457; doi:10.1371/journal.pone.0181501)
Supplement: S1 Fig — tetA amplified from pBBR1MCS3 was linked to three different promoters: the tetA gene native promoter (PtetA) [32], synthetic constitutive promoter CP25 (PCP25) [35], or the P3 promoter including a bicistronic ribosome binding site termed P3-BCD2 (PP3BCD2) [36], to construct the three different dual selection cassettes PtetA-TetA, PCP25-TetA, and PP3BCD2-TetA, respectively. These cassettes were individually transformed into MG-Red to construct dual selection cassette-variant strains, MG-PT, MG-PC, and MG-PP3. Detailed information is provided in the text. (PDF) [file pone.0181501.s001.pdf]

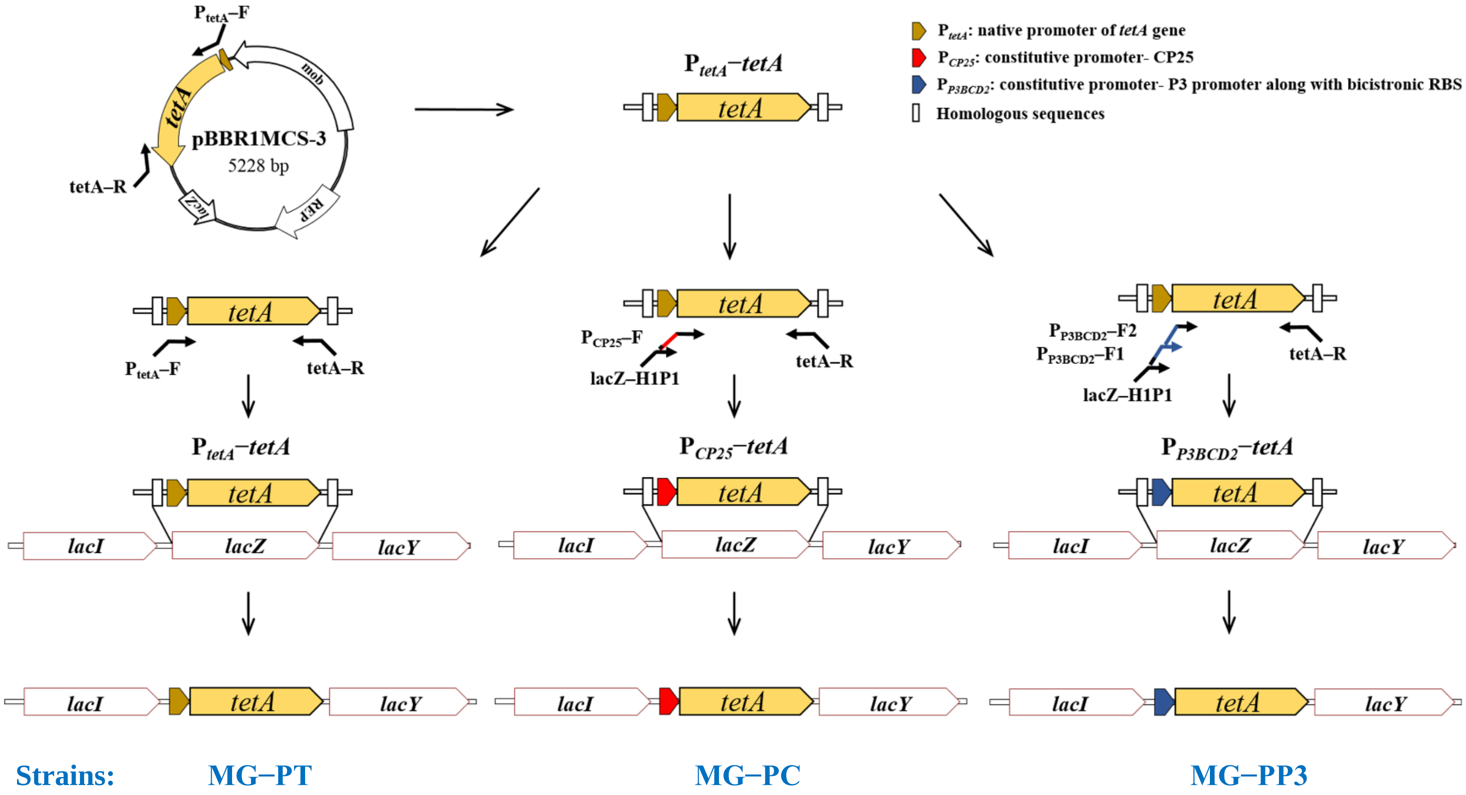

S1 Fig. Construction of *tetA* dual selection cassettes with three different promoters. *tetA* amplified from pBBR1MCS3 was linked to three different promoters: the *tetA* gene native promoter (P_{tetA}) [32], synthetic constitutive promoter CP25 (P_{CP25}) [35], or the P3 promoter including a bicistronic ribosome binding site termed P3-BCD2 (P_{P3BCD2}) [36], to construct the three different dual selection cassettes P_{tetA}-TetA, P_{CP25}-TetA, and P_{P3BCD2}-TetA, respectively. These cassettes were individually transformed into MG-Red to construct dual selection cassette-variant strains, MG-PT, MG-PC, and MG-PP3. Detailed information is provided in the text.
