# Supplementary material for: Oligo- and dsDNA-mediated genome editing using a tetA dual selection system in Escherichia coli
Source: PLoS One. 2017 Jul 18;12(7):e0181501. doi: 10.1371/journal.pone.0181501 (PMC5515457; doi:10.1371/journal.pone.0181501)
Supplement: S2 Fig — (A) PtetA: native promoter of tetA [32]; (B) PCP25: synthetic constitutive promoter CP25 [35]; (C) PP3BCD2: synthetic constitutive promoter P3 including bicistronic ribosome binding site [36]; P1 and P4: priming sequences [47]; H1 and H2: upstream and downstream homologous sequences; −35 and −10: promoter regions; +1: transcription start site. (PDF) [file pone.0181501.s002.pdf]

A P_{tetA}–*tetA*

TATGTTGTGTGGAATTGTGAGCGGATAACAATTTCACACAGGAAACAGCTGTGTAGGCTGGAGCTGCTTCGCAATTCTCATGTTTGACAGCTTATCATCGATAAG

LacZ–H1 P1 -35

CTTTAATGCGGTA**G**TTTATCACAGTTAAATTGCTAACGCAGTCAGGCACCGTGT**ATG····*tetA*····TAG**GGTCGACGGATCCCCGGAATTAATAACCGGGCAGGCCATGT

-10 +1 RBS P4 LacZ–H2

CTGCCCGTATTTCGCGTAAGGAAATCCAT

B P_{CP25}–*tetA*

TATGTTGTGTGGAATTGTGAGCGGATAACAATTTCACACAGGAAACAGCTGTGTAGGCTGGAGCTGCTTCGCTTTGGCAGTTTATTCTTGACATGTAGTGAGGG

LacZ–H1 P1 -35

GGCTGGTATAATCACATA**G**TACTGTTCACACAGGAAACAGCT**ATG····*tetA*····TAG**GGTCGACGGATCCCCGGAATTAATAACCGGGCAGGCCATGTCTGCCCGT

-10 +1 RBS P4 LacZ–H2

ATTTCGCGTAAGGAAATCCAT

C P_{P3BCD2}–*tetA*

TATGTTGTGTGGAATTGTGAGCGGATAACAATTTCACACAGGAAACAGCTGTGTAGGCTGGAGCTGCTTCGAAAAAATTTATTTGCTTATTAATCATCCGGCTCG

LacZ–H1 P1 -35

TATAATGTG**T**GGAGGGCCCAAGTTCACTTAAAAAGGAGATCAACAATGAAAGCAATTTTCGTACTGAAACATCTTAATCATGCTAAGGAGGTTTTCTA**ATG····*tetA***

-10 +1 RBS1 RBS2

····TAGGGTCGACGGATCCCCGGAATTAATAACCGGGCAGGCCATGTCTGCCCGTATTTCGCGTAAGGAAATCCAT

P4 LacZ–H2

S2 Fig. Nucleotide sequences of the promoters used for chromosomal *tetA* expression in the *lacZ* region. (A) P_{tetA}: native promoter of *tetA* [32]; (B) P_{CP25}: synthetic constitutive promoter CP25 [35]; (C) P_{P3BCD2}: synthetic constitutive promoter P3 including bicistronic ribosome binding site [36]; P1 and P4: priming sequences [47]; H1 and H2: upstream and downstream homologous sequences; −35 and −10: promoter regions; +1: transcription start site.
