# Supplementary material for: Oligo- and dsDNA-mediated genome editing using a tetA dual selection system in Escherichia coli
Source: PLoS One. 2017 Jul 18;12(7):e0181501. doi: 10.1371/journal.pone.0181501 (PMC5515457; doi:10.1371/journal.pone.0181501)
Supplement: S3 Fig — (A) Agarose gel electrophoresis of the PCR products following the insertion of gfp downstream of dxs (B) Steps and primers (dxs-seqF2 and dxs-seqR2) used to confirm recombination. The size of specific PCR products is given in parentheses. (PDF) [file pone.0181501.s003.pdf]

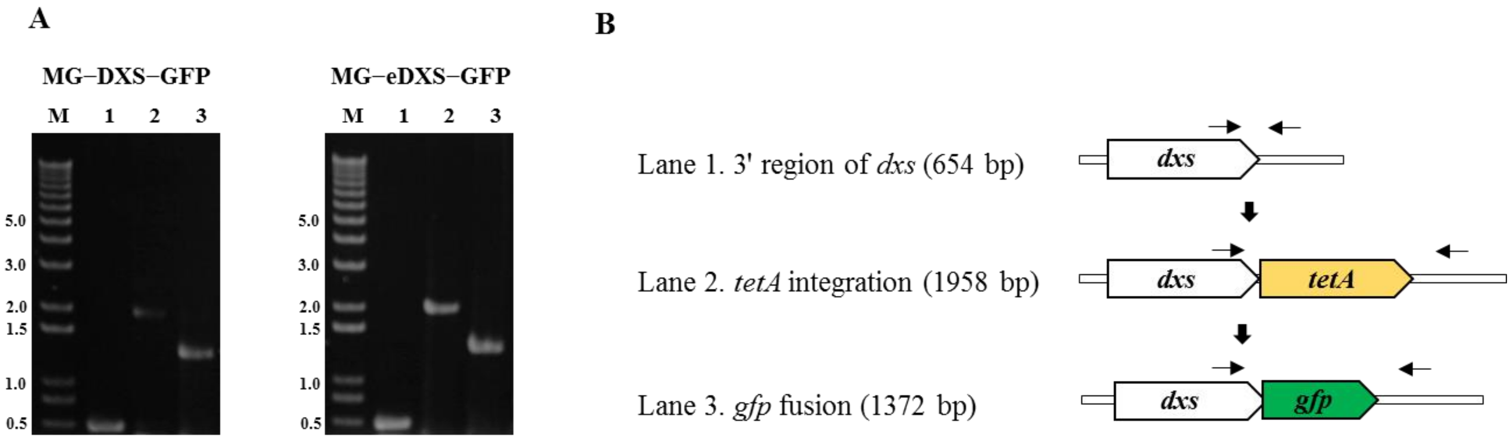

S3 Fig. Gene insertion using dsDNA-homologous recombination. (A) Agarose gel electrophoresis of the PCR products following the insertion of *gfp* downstream of *dxs* (B) Steps and primers (dxs-seqF2 and dxs-seqR2) used to confirm recombination. The size of specific PCR products is given in parentheses.
