# Supplementary material for: Oligo- and dsDNA-mediated genome editing using a tetA dual selection system in Escherichia coli
Source: PLoS One. 2017 Jul 18;12(7):e0181501. doi: 10.1371/journal.pone.0181501 (PMC5515457; doi:10.1371/journal.pone.0181501)
Supplement: S1 Table — (PDF) [file pone.0181501.s004.pdf]

S1 Table. Timeline of the workflow for the *tetA* dual selection.

	**CRISPR** [27]	***tetA-sacB* dual selection** [15]	***SceI* counter-selection** [6]	***tetA* dual selection**
Day 1	1) Transform pCas9cr4<br>2) Clone spacer	Transform λ-Red plasmid	1) Transform λ-Red plasmid<br>2) Clone Mutation Cassette	Transform λ-Red plasmid
Day 2	Grow clones	Grow cells overnight	Screen and sequence clones	Grow cells overnight
Day 3	Isolate plasmid and transform into cells with pCas9cr4	1) Subculture and induce λ-Red<br>2) Transform *tetA-sacB* cassette	PCR amplify Mutation Cassette and Transform	1) Subculture and induce λ- Red<br>2)Transform*tetA* dual cassette
Day 4	1) Start culture and induce λ-Red<br>2) Transform linear DNA, induce Cas9	Restreak colonies on counter-selection medium	Restreak colonies	1) Confirm *TetA* integration in Tet^R by PCR<br>2) Start overnight culture
Day 5	1) Screen colonies<br>2) Grow at 37° C to cure pKDsgRNA[4]	Identify $sucrose^s$ clones and start overnight culture	Resuspend colonies and plate to induce DSB	1) Induce λ-Red and transform oligo or dsDNA<br>2) Subculture in Ni negative selection media for overnight at 37°C
Day 6	Transform pKD-p15, induce Cas9	1) Start culture and induce λ-Red<br>2) Transform with linear DNA and counterselection medium	Patch colonies to screen for Ab^S	Streak on LB and incubate for overnight at 37°C
Day 7	Patch colonies for cm^s, Grow at 37° C	Incubate at 42° C[*]	Passage cells to cure plasmid	1) Screen by PCR<br>2) Plasmid free colonies<br>OR<br>1) Replica on LB and Tet-LB agar[@]
Day 8	Plasmid free colonies	Screen by PCR or Tet^S	Plasmid free colonies	From replica plates, select cells grown only on LB and screen by PCR[@]
1 mutation w/ curing	8 Days[#]	8 Days	8 Days[#]	7~8 Days

[*] It is recommended to incubate the cells at 42°C for at least 2 days for full colony formation. Thereby it would increase one more day.
[#]These methods require prior plasmid cloning. It would increase the time to additional 5 -7 days.
[@]Replica plating is optional to get 100% recombinants from enriched transformants.
The table was adapted from Reisch and Prather[27].
